# IgE reactivity to α-Gal in relation to Lyme borreliosis

**DOI:** 10.1371/journal.pone.0185723

**Published:** 2017-09-27

**Authors:** Ivar Tjernberg, Carl Hamsten, Danijela Apostolovic, Marianne van Hage

**Affiliations:** 1 Department of Clinical Chemistry and Transfusion Medicine, Kalmar County Hospital, Kalmar, Sweden; 2 Department of Clinical and Experimental Medicine, Linköping University, Linköping, Sweden; 3 Department of Medicine Solna, Immunology and Allergy Unit, Karolinska Institutet and University Hospital, Stockholm, Sweden; Johns Hopkins University, UNITED STATES

## Abstract

**Background:**

An association between tick bites, the development of immunoglobulin E (IgE) antibodies to galactose-α-1, 3-galactose (α-Gal) and red meat allergy has recently been reported. Here we wanted to elucidate the relation between tick exposure, IgE antibodies to α-Gal and Lyme borreliosis (LB).

**Methods:**

In the highly LB endemic area of Kalmar County, Sweden, serum samples and health inquiries from 518 blood donors were included. All sera were investigated for multiple IgG anti-*Borrelia* antibodies using a multiplex assay (recomBead, Mikrogen). In addition, three serially collected sera over a six month period from 148 patients with clinically defined erythema migrans (EM) were included. IgE antibodies against α-Gal were determined using ImmunoCAP (Thermo Fisher Scientific).

**Results:**

In blood donors reporting previous LB (n = 124) IgE to α-Gal was found in 16%, while in donors denying previous LB but with multiple anti-*Borrelia* antibodies (n = 94; interpreted as asymptomatic LB) 10% were IgE α-Gal-positive. Finally, in donors without *Borrelia* antibodies denying previous LB (n = 300) 14% showed IgE to α-Gal. No significant difference in proportions among the groups were found. In EM patients, IgE to α-Gal was found in 32/148 (22%) at diagnosis, 31/148 (21%) after two-three months and 23/148 (16%) after six months. A significant reduction of proportion and level of IgE to α-Gal was found between the second and third sample (p<0.01). A positive IgE anti α-Gal was more common among men compared with women both in blood donors and in EM patients (p≤0.01).

**Conclusions:**

IgE to α-Gal reactivity was common in a tick endemic area but showed no significant relation to previous LB. IgE anti-α-Gal reactivity in EM patients peaked within three months of diagnosis of EM, after which it waned indicating that recent tick exposure is of importance in α-Gal sensitization. Furthermore, IgE anti α-Gal was more common in men compared with women.

## Introduction

In the last few years, an association between immunoglobulin E (IgE) antibodies directed towards the mammalian carbohydrate galactose-α-1, 3-galactose (α-Gal) and red meat allergy has been described [1–4]. The anti-α-Gal response was first identified when a subset of patients treated with cetuximab (a chimeric mouse human monoclonal antibody targeting the epidermal growth factor) developed severe anaphylactic reactions at the first treatment [5]. Van Nunen and colleagues from Australia then documented an association between large local reactions to bites from the tick *Ixodes (I*.*) holocyclus* and adult onset red meat allergy and hypothesized a pathogenic relationship [6]. Later, Platts-Mills and co-workers from North America reported that the development of this specific antibody may be linked to the exposure of tick bites by the tick species *Amblyomma americanum* [7]. We have recently identified α-Gal in the gastrointestinal tract of the principal European vector of Lyme borreliosis (LB), the tick *Ixodes* (*I*.) *ricinus* [8]. Furthermore, α-Gal has also shown to be present in saliva of the ticks *Haemaphysalis longicornis* and *Ambylomma sculptum* [9, 10]. This and recent data about the prevalence of sensitization to α-Gal [11, 12], strengthens the association between tick bites of various species and the development of IgE antibodies to α-Gal and in turn red meat allergy [8, 13]. In addition, as α-Gal is structurally related to the blood group B antigen, a protective effect of blood group B on the development of IgE anti-α-Gal and red meat allergy has been reported [2, 14]. However, tick exposure is very common in many geographic areas including the south of Sweden and also in particular the Åland Islands in the Baltic Sea [15], but cases of red meat allergy are few in comparison [2]. Thus, determinants of which of the tick bitten individuals that will develop IgE anti-α-Gal antibodies are still unknown. Similarly, which of the IgE α-Gal-positive individuals that will develop clinical red meat allergy remains to be shown, although the level of IgE anti-α-Gal seems to be of importance with cases of red meat allergy showing significantly higher IgE levels compared to α-Gal positive healthy blood donors [2]. In parallel with red meat allergy, the risk of developing LB after a tick bite has been demonstrated to be small in Scandinavia, although *Borrelia* has been identified in some 26% of ticks [15, 16]. LB is the most common known tick borne infection in both Europe and North America, an infection that may present with various clinical signs and symptoms. The skin manifestation erythema migrans (EM) is the most common clinical manifestation of LB with a frequency of at least 70% of all clinical LB [17, 18]. Interestingly, an asymptomatic *Borrelia* infection, defined as a *Borrelia* IgM/IgG antibody seroconversion after tick bite in the absence of clinical symptoms, seems to be as common as developing clinical LB [15]. Our previous work revealed that 22% of patients with LB in the Stockholm area have positive IgE levels to a-Gal at significantly higher frequency than healthy blood donors [2].

In this study, we investigated the IgE reactivity against α-Gal and *I*. *ricinus* in relation to *Borrelia* IgG antibody status, blood group status, self-reported tick exposure and previous LB in Swedish blood donors collected in a LB endemic area, enabling the discrimination of previous clinical LB and asymptomatic LB. Furthermore, we wanted to elucidate the IgE anti-α-Gal status in patients with EM in acute phase as well as in convalescent samples in order to determine the role of time in relation to IgE anti-α-Gal.

## Material and methods

### Study subjects

#### Blood donors

A total of 1126 healthy blood donors in Kalmar County in Sweden were consecutively included in an adjacent clinical study regarding asymptomatic LB in the spring of 2012, when scheduled for routine blood donation. Blood donor sera were collected together with health inquires including information on previous tick exposure and previous history of LB. A complete set of health inquiry and blood sera was obtained from 1113 blood donors. Blood donors were divided in three groups according to whether they answered yes, no or undetermined to previous history of LB. To ensure a higher level of specificity, blood donors answering yes to previous history of LB were also required to have a clinical diagnosis to be included in the group.

All blood donor sera were screened for specific IgG antibodies against 13 different borrelial antigens using a commercial multiplex kit, recomBead Borrelia IgG (Mikrogen GmbH, Neuried, Germany). Analyses were performed according to the manufacturer’s instructions, whereby the results of the individual antigen reactivities were added to a final sum using a scoring system. A total sum of eight points and more were regarded as a positive test result, according to the manufacturer’s instructions.

The following selection criteria were used for selection of three subgroups of blood donors investigated in this study: All blood donors reporting previous clinical LB confirmed by medical doctor’s diagnosis, n = 124 (previous LB). All blood donors denying previous LB but with a positive test result in the multiplex *Borrelia* IgG antibody test, n = 94 (previous asymptomatic LB). Finally, a group of blood donors, n = 300, denying previous LB and with a negative anti-*Borrelia* antibody test (no known previous *Borrelia* exposure) was included in the following manner: The first 30 consecutively included blood donors of women and men in the following age groups were selected; 18–29, 30–39, 40–49 and 50–59 years of age. Regarding the age group of 60 years and more only 22 women were available, therefore an additional eight men were included in this age group, thereby consisting of 38 men. In total, 518 blood donors in different subgroups with serum samples and complete health inquiry results were included in this study. The blood donor serum samples had been stored at −20°C and freeze-thawed once prior to the analyses in this study.

Blood group results, AB0 and RhD, were collected from the blood donor journal system ProSang 2008 (Databyrån, Stockholm, Sweden), and the expected AB0 blood group distribution nationally was obtained from official data [19].

#### Erythema migrans patients

In a separate prospective study of patients with LB in Kalmar County in Sweden during 2003, clinical data was recorded in a study protocol and serum samples were drawn at the first consultation, after two to three months and finally after six months. EM was diagnosed by a medical doctor by typical clinical skin features and >5 cm in diameter. All EM serum samples were initially analysed for anti-*Borrelia* antibodies using enzyme-linked immunosorbent assay detecting C6 peptide antibodies (non-resolving assay for IgM and IgG antibodies) with analyses performed and results interpreted according to the manufacturer (optical density value >0.15) (Immunetics^®^, Boston, MA, USA). Serum samples from a total of 148 EM patients, each with a set of three serum samples, were available for this study [20, 21]. The serum samples from EM patients had been stored at −20°C and freeze-thawed twice before the analyses in this study.

### IgE assays

IgE antibodies against α-Gal were determined by ImmunoCAP (Phadia AB/Thermo Fisher Scientific, Uppsala, Sweden) for all included blood donors as well as EM patients at all three time points. Blood donor samples and EM samples IgE positive to α-Gal (≥ 0.1 kU_A_/L) were further investigated for IgE against *I*. *ricinus* using 5 mg of biotinylated *I*. *ricinus* antigen coupled to Streptavidin ImmunoCAP as previously described [2, 8]. Total IgE and Phadiatop^®^, a mix of common inhalant allergens, (ImmunoCAP, Phadia AB/Thermo Fisher Scientific) assays were also performed in the first serum sample of all EM patients in order to assess atopy. IgE levels ≥ 0.1 kU_A_/L against *I*. *ricinus* and ≥0.35 kU_A_/L against Phadiatop^®^ were considered positive. All IgE analyses were performed in accordance with the instructions of the manufacturer at the Department of Clinical Immunology, Karolinska University Hospital Solna, Stockholm, Sweden.

### Statistics and ethics

Statistical analyses were performed using Fisher’s exact two-tailed test for proportions and the non-parametric Mann-Whitney’s U-test for comparing age between groups. An exact test corresponding to McNemar’s paired chi-squared modification, first proposed by Liddell (1983) was used for paired measurements of proportions [22]. The Friedman ANOVA and Wilcoxon paired test were used when comparing IgE anti-α-Gal seropositivity levels in EM patients over time (Statistica 12). Comparison of total IgE and anti-α-Gal specific IgE was performed with Spearman rank correlation using Prism 7 (GraphPad Software, La Jolla, Calif). A *p*<0.05 was considered statistically significant. The study was approved by the regional ethical review board of Linköping University, Sweden. Written consent was obtained from the study participants.

## Results

### Clinical features

Descriptive data regarding sex and age distributions and self-reported tick exposure among all studied blood donors taken together (n = 518) and divided into the three identified subgroups (donors with previous LB, donors previously asymptomatically *Borrelia* infected and donors without known *Borrelia* exposure) are shown in S1 Table. In 105 of 518 (20%) blood donors the average number of tick bites annually, counting the five last years preceding the inclusion in the study in 2012, was reported to be five bites or more. Self-reported tick bites of at least five bites or more annually did not differ between women 52/234, 22% and men 53/284, 19% (data missing for two men), *p* = non-significant. Corresponding data regarding sex, age, duration of EM at diagnosis and self-reported previous LB for the EM patients are shown in S2 Table (n = 148). The median duration of EM at diagnosis, i.e. at the first serum sampling time, was seven days. In 46/148 (31%) of the EM patients at least one previous episode of LB was reported and they were assessed as subjects exposed to LB re-infection.

### Allergen-specific IgE

#### Blood donors

The prevalence of IgE reactivity to α-Gal among the blood donors was 14% (71/518) with a median level of 0.30 kU_A_/L (range 0.10–17 kU_A_/L). Divided into sub-groups according to previous *Borrelia* status, the rate of IgE positivity to α-Gal varied between 10–16% (Table 1). A comparison between blood donors IgE positive and negative to α-Gal revealed a male dominance among IgE positive donors, p = 0.01 (Table 2). No significant difference in tick exposure, previous LB or anti-*Borrelia* antibody status was shown comparing IgE anti-α-Gal positive and negative donors. In 22 of the 71 (31%) IgE α-Gal-positive samples a positive (≥0.1 kU_A_/L) level of IgE to *I*. *ricinus* was found, with a median level of 0.54 kU_A_/L (range 0.10–3.8 kU_A_/L).

**Table 1:** IgE anti-α-Gal positive rate in blood donors according to *Borrelia* status.

	IgE α-Gal positive (≥ 0.1 kU_A_/L), numbers (%)
All blood donors (n = 518)	71 (14)
Donors with previous LB (n = 124)	20 (16)
Donors previously asymptomatically *Borrelia* infected (n = 94)	9 (10)
Donors without known *Borrelia* exposure^a^ (n = 300)	42 (14)

n = numbers

LB = Lyme borreliosis

^a^ Donors denying previous LB also lacking serum anti-*Borrelia* antibodies

**Table 2:** Basic clinical data on blood donors according to IgE anti-α-Gal status.

	Female/Male, numbers (% females)	Median age, (range in years)	Selfreported tick exposure > 5 bites annually, numbers (%)	Previous LB, numbers (%)	Positive anti-*Borrelia* antibody test^a^, numbers (%)
IgE α-Gal positive (≥ 0.1 kU_A_/L) blood donors (n = 71)	22/49^b^ (23)	53 (18–69)	15^c^ (21^c^)	20 (28)	11 (15)
IgE α-Gal negative (<0.1 kU_A_/L) blood donors (n = 447)	212/235^b^ (47)	46 (18–72)	90^c^ (20^c^)	104 (23)	103 (23)

LB = Lyme borreliosis

n = numbers

^a^ recomBead Borrelia IgG

^b^ p = 0.01

^c^ Data missing on one blood donor

Blood group distributions among the blood donors according to IgE anti-α-Gal status are shown in S3 Table including the expected distribution of blood groups in the population in Sweden for comparison [19]. Among blood donors IgE positive to α-Gal, five (7.0%) were blood group B (including blood groups B+, AB+, B− and AB−) compared to α-Gal-negative blood donors where 60/447 (13%) were B-positive (p = 0.18). Median positive IgE reactivity to α-Gal among blood group B was 0.87 kU_A_/L and among B-negative 0.32 kU_A_/L (p = 0.50).

#### Erythema migrans patients

**Fig 1:**
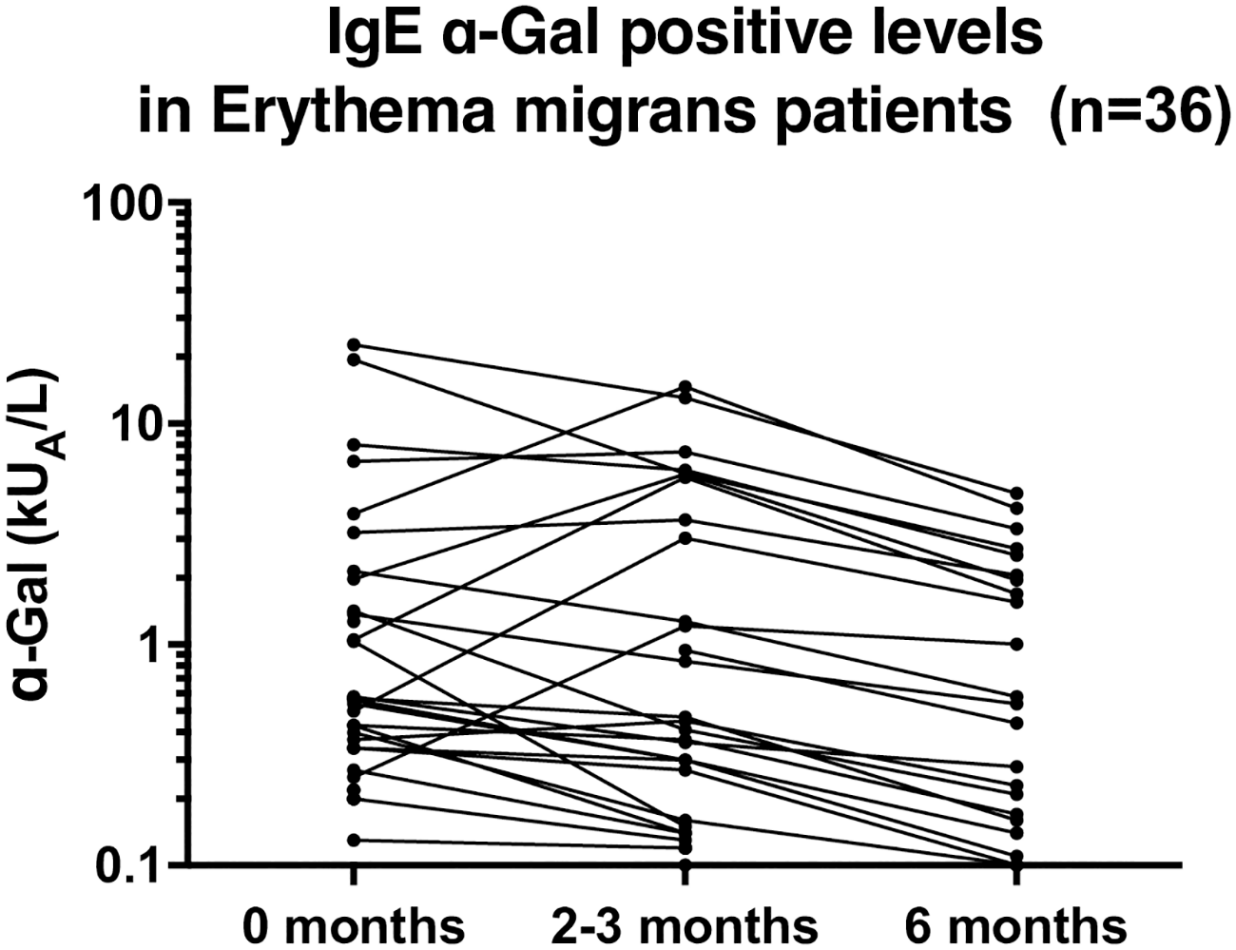
IgE levels against α-Gal at the three different time points (at diagnosis=“0 months”; after two to three months and six months) among the 36 α-Gal IgE positive erythema migrans patients. The levels were compared between the different time points. A significant reduction in IgE anti-α-Gal levels from both the first and second sample to the third sample (p<0.01).

A total of 36/148 (24%) EM patients were IgE positive to α-Gal in at least one of the three samples. At the EM diagnosis, 32 of 148 (22%) patients were IgE positive (≥ 0.1 kU_A_/L) to α-Gal, at two to three months 31/148 (21%) and finally 23/148 (16%) were IgE positive at six months after diagnosis, Table 3. Comparing levels of IgE anti-α-Gal among those 36 individuals positive in at least one sample, a significant decrease was noted between the first and third sample as well as between the second and third samples (*p* < 0.01), Fig 1. The difference was also confirmed by paired measurement of proportions between the second and third sample Table 3 (*p* < 0.01). In Table 3 proportions of IgE to *I*. *ricinus*-positive samples among IgE anti-α-Gal-positive samples are also given together with levels of total IgE and the Phadiatop positive rate (26/148; 18%) for EM patients examined at diagnosis.

**Table 3:** IgE anti-α-Gal positive, total IgE and Phadiatop status in erythema migrans patients.

	Erythema migrans patients (n = 148)			p-value^a^
	At diagnosis, numbers (%)	2–3 months, numbers (%)	6 months, numbers (%)	
IgE α-Gal-positive (≥ 0.1 kU_A_/L)	32 (22)	31 (21)	23 (16)	<0.01
IgE α-Gal-positive samples, median reactivity (range) (kU_A_/L)	0.55 (0.13–23)	0.41 (0.10–15)	0.54 (0.10–4.8)	
IgE to *Ixodes ricinus* (≥ 0.1 kU_A_/L) among IgE α-Gal-positive samples	10/32^b^ (31^b^)	13/31 (42)	11/23 (48)	
IgE to *Ixodes ricinus*-positive samples, median reactivity (range) (kU_A_/L)	0.21 (0.14–1.6)	0.26 (0.14–8.1)	0.20 (0.12–3.5)	
Total-IgE, median (range) (kU_A_/L)	30 (2.5–845)^c^			
Phadiatop positive (≥0.35 kU_A_/L)	26^d^ (18^d^)			

n = numbers

^a^ No significant difference at diagnosis compared with 2–3 months, but significant reduction between 2–3 months and 6 months, paired measurement

^b^ One sample missing

^c^ Nine samples missing

^d^ Eight samples missing

**Fig 2:**
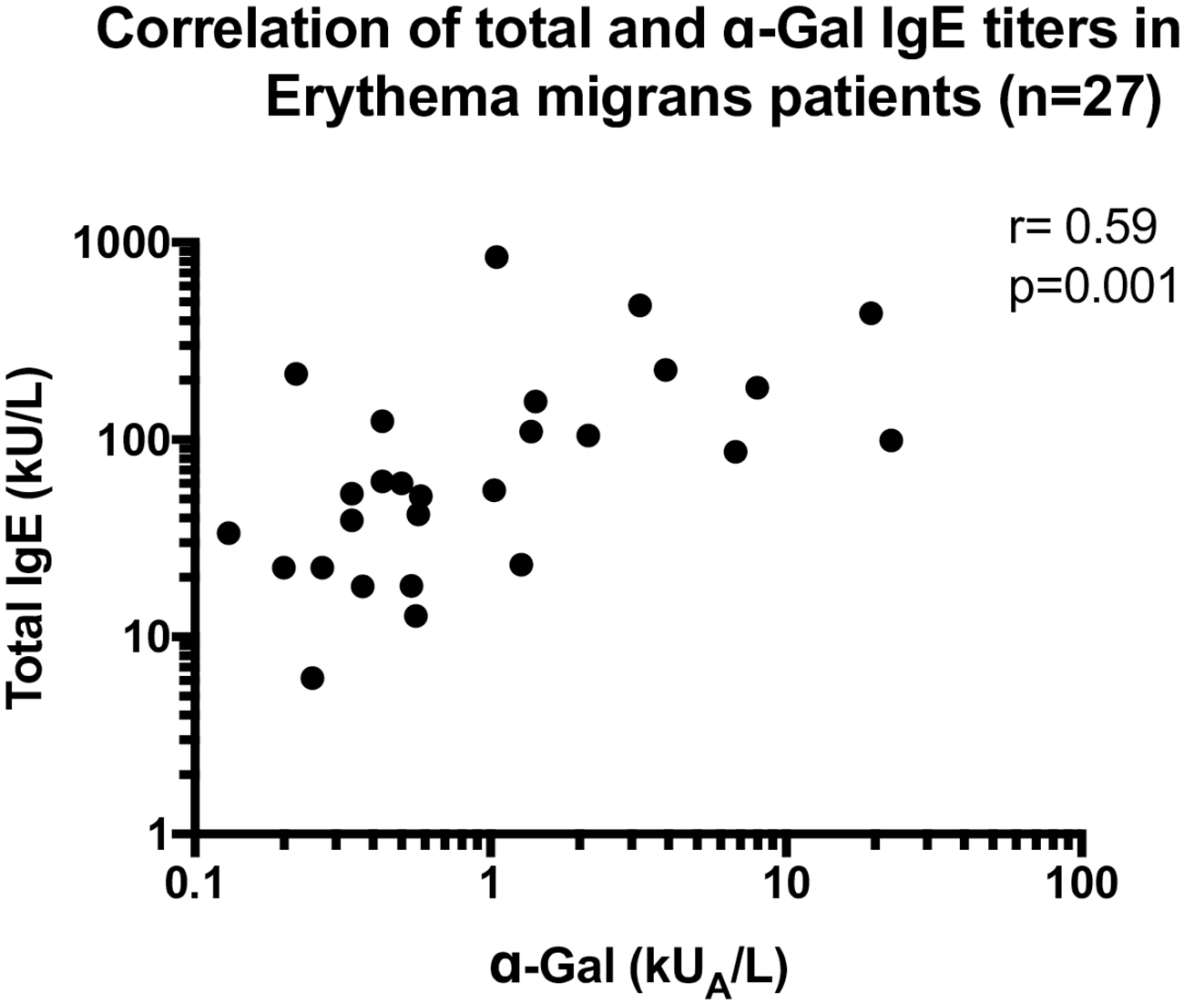
Correlation between total IgE and IgE responses to α-Gal in erythema migrans patients (n = 27).

EM patients were also divided according to IgE anti-α-Gal status, see Table 4. A positive IgE anti-α-Gal result was more common among EM men compared with women (*p* < 0.001), similar to the results found in blood donors, Table 2. No significant differences were shown comparing median age, Phadiatop status, anti-*Borrelia* antibody status or reported previous LB between IgE anti-α-Gal positive and negative EM patients (Table 4). In addition, the individual IgE anti-α-Gal levels in the 36 EM patients IgE positive to α-Gal at any sample time are shown in Fig 1. Finally, a significant positive correlation between total IgE and IgE anti-α-Gal levels was noted (Fig 2).

**Table 4:** Comparison of IgE α-Gal positive and negative erythema migrans patients.

	IgE α-Gal positive erythema migrans patients at diagnosis (n = 32), numbers (%)	IgE α-Gal negative erythema migrans patients at diagnosis (n = 116), numbers (%)	p-value
Female/Male	11/21 (34/66)	79/37 (68/32)	<0.001^a^
Median age, range (years)	58 (7–78)	58 (16–84)	NS^b^
Phadiatop IgE positive	6^c^ (19^c^)	20^c^ (17^c^)	NS^a^
Anti-*Borrelia* antibody positive	7 (22)	46 (40)	0.09^a^
Previous LB	5^d^ (16^d^)	41^e^ (35^e^)	0.076^a^

n = numbers

LB = Lyme borreliosis

^a^ Fisher's exact two-tailed test

^b^ Mann-Whitney U-test

^c^ Four samples missing

^d^ Unknown for four patients

^e^ Unknown for one patient

## Discussion

The present study of a large number of sera from blood donors sub-grouped according to previous LB status and EM patients followed over time shows that IgE anti-α-Gal is commonly found in an LB endemic area with a frequency of 10–22% with a male predominance. Furthermore, the level of IgE anti-α-Gal in α-Gal positive cases decreases over time in EM patients.

Previously, 10% of blood donors in the Stockholm area in Sweden have been shown to be IgE positive to α-Gal, thus in the same order of magnitude as found in this study with 14% [2]. For further comparison in the general population, IgE to α-Gal was found in 5.5% in Denmark and in 8.1% in Spain [12]. Considering the variation in tick abundance over different regions and geographical areas in Europe, variations in IgE anti-α-Gal reactivity rates are to be expected [23]. However, although tick exposure seems to be an important risk factor for developing IgE anti-α-Gal, sensitization may also be related to cat ownership [12]. In the present materials investigated, no data regarding pet ownership was available. In Sweden, the highest incidence of LB has been reported in Kalmar County, the county in which the samples in this study were collected [24]. As there is no systematically gathered data regarding tick exposure in the general population in Sweden, LB incidence may indicate tick prevalence to some extent. Thus, we would expect high rates of IgE α-Gal sensitization in Kalmar County in Sweden, and the 14% we found seems to be in line with this reasoning. However, LB incidence is of course also affected by a number of other variables including *Borrelia* abundance in ticks as well as behaviour regarding activities associated with tick exposure and tick removal routines in the population. Therefore, the interpretation of sensitization in relation to tick exposure should be made with caution. Interestingly, a considerable proportion of the blood donors in this study reported frequent tick bites, but no difference in the proportion of multiple tick bites annually was found comparing IgE α-Gal negative with positive donors. One possible explanation for this may be the sampling time; the blood donor samples were collected during the winter with an expected low tick activity in the environment. Thus, tick exposure in the blood donor group generally consisted of exposure the previous summer-autumn and historically. In contrast for the EM group, a recent tick bite must have taken place in order for the EM to occur. Therefore, our data suggests that recent tick exposure may give rise to α-Gal sensitization with decreasing titers over time approaching the general sensitization level in the population of that geographical area. In this study, 22% of the EM patients showed positive IgE levels to α-Gal at diagnosis, in fact the same level as reported from the Stockholm area [2]. Regarding sensitization depending on previous symptomatic or asymptomatic LB, no significant difference was shown. Thus, this does not seem to be a factor that determines which individuals that will develop IgE anti-α-Gal.

In addition to the association between IgE anti-α-Gal and timing of tick exposure, we found more men than women to be sensitized to α-Gal both among blood donors and EM patients. The significance of that finding remains to be elucidated, but a similar difference was also shown in Denmark but not in Spain [12]. The same sex difference has also been shown for anti-*Borrelia* antibodies, and may reflect a difference in tick exposure and removal strategies between men and women [25]. Data suggests that men may remove ticks later than women thus permitting greater time for exposure to tick antigens and pathogens [15].

In harmony with previous data, where a significant correlation between IgE anti-α-Gal and total IgE levels among red meat allergic patients was shown, a similar correlation was observed in this study [2]. Whereas the majority of patients with red meat allergy also showed IgE antibodies to *I*. *ricinus*, we could only demonstrate that co-sensitization in some 31–48% of the IgE anti-α-Gal-positive EM patients and blood donors. However, it is important to bear in mind that no data regarding red meat allergy was available in the present material in contrast to the study by Hamsten *et al*. in which red meat allergy patients were investigated.

Previously it has been shown that blood group B protects from red meat allergy, however it does not seem to inhibit from sensitization to α-Gal [2]. In the study by Hamsten *et al*. 13% of α-Gal-positive blood donors were blood group B and 22% of patients with a positive *Borrelia* serology. In our study 7% of α-Gal-positive blood donors were blood group B, thus in line with previous findings.

The strengths of this study include the prospective design and collection of patients and blood donors together with standardised inquiry data, the sample size and clinical data regarding LB including the serially followed EM patients. The major limitation being the lack of data regarding red meat reactions and allergy.

## Conclusions

In conclusion, this study confirms that IgE reactivity to α-Gal is commonly found in blood donors in an LB endemic area, but with no significant relation to previous LB, self-reported tick exposure or *Borrelia* antibody status. However, the IgE response to α-Gal showed a time dependent pattern in EM patients with reactivity peaking within three months of diagnosis, then declining. This indicates that recent tick exposure is of importance in sensitization to α-Gal. Furthermore, IgE anti-α-Gal was more common in men compared with women in both blood donors and EM patients, but the significance of this finding in relation to red meat allergy remains to be shown.

## Supporting information

S1 Table Basic descriptive data on blood donors and subgroups according to Borrelia status. (XLSX)

S2 Table Basic descriptive data on erythema migrans patients. (XLSX)

S3 Table Blood group distribution in blood donors according to IgE anti-α-Gal status. (XLSX)

## References

[1] Commins SP, Satinover SM, Hosen J, Mozena J, Borish L, Lewis BD, et al Delayed anaphylaxis, angioedema, or urticaria after consumption of red meat in patients with IgE antibodies specific for galactose-alpha-1,3-galactose. The Journal of allergy and clinical immunology. 2009;123(2):426–33. Epub 2008/12/17. doi: 10.1016/j.jaci.2008.10.052 . 19070355 10.1016/j.jaci.2008.10.052 PMC3324851

[2] Hamsten C, Tran TA, Starkhammar M, Brauner A, Commins SP, Platts-Mills TA, et al Red meat allergy in Sweden: association with tick sensitization and B-negative blood groups. The Journal of allergy and clinical immunology. 2013;132(6):1431–4. doi: 10.1016/j.jaci.2013.07.050 . 24094548 10.1016/j.jaci.2013.07.050 PMC4036066

[3] Morisset M, Richard C, Astier C, Jacquenet S, Croizier A, Beaudouin E, et al Anaphylaxis to pork kidney is related to IgE antibodies specific for galactose-alpha-1,3-galactose. Allergy. 2012;67(5):699–704. Epub 2012/04/13. doi: 10.1111/j.1398-9995.2012.02799.x . 22494361 10.1111/j.1398-9995.2012.02799.x

[4] Mullins RJ, James H, Platts-Mills TA, Commins S. Relationship between red meat allergy and sensitization to gelatin and galactose-alpha-1,3-galactose. The Journal of allergy and clinical immunology. 2012;129(5):1334–42 e1. doi: 10.1016/j.jaci.2012.02.038 . 22480538 10.1016/j.jaci.2012.02.038 PMC3340561

[5] Chung CH, Mirakhur B, Chan E, Le QT, Berlin J, Morse M, et al Cetuximab-induced anaphylaxis and IgE specific for galactose-alpha-1,3-galactose. N Engl J Med. 2008;358(11):1109–17. Epub 2008/03/14. doi: 10.1056/NEJMoa074943 . 18337601 10.1056/NEJMoa074943 PMC2361129

[6] Van Nunen SA, O'Connor KS, Clarke LR, Boyle RX, Fernando SL. An association between tick bite reactions and red meat allergy in humans. Med J Aust. 2009;190(9):510–1. . 19413526 10.5694/j.1326-5377.2009.tb02533.x

[7] Commins SP, James HR, Kelly LA, Pochan SL, Workman LJ, Perzanowski MS, et al The relevance of tick bites to the production of IgE antibodies to the mammalian oligosaccharide galactose-alpha-1,3-galactose. The Journal of allergy and clinical immunology. 2011;127(5):1286–93 e6. Epub 2011/04/02. doi: 10.1016/j.jaci.2011.02.019 . 21453959 10.1016/j.jaci.2011.02.019 PMC3085643

[8] Hamsten C, Starkhammar M, Tran TA, Johansson M, Bengtsson U, Ahlen G, et al Identification of galactose-alpha-1,3-galactose in the gastrointestinal tract of the tick Ixodes ricinus; possible relationship with red meat allergy. Allergy. 2013;68(4):549–52. Epub 2013/02/19. doi: 10.1111/all.12128 . 23414348 10.1111/all.12128

[9] Araujo RN, Franco PF, Rodrigues H, Santos LC, McKay CS, Sanhueza CA, et al Amblyomma sculptum tick saliva: alpha-Gal identification, antibody response and possible association with red meat allergy in Brazil. Int J Parasitol. 2016;46(3):213–20. doi: 10.1016/j.ijpara.2015.12.005 . 26812026 10.1016/j.ijpara.2015.12.005 PMC5523130

[10] Chinuki Y, Ishiwata K, Yamaji K, Takahashi H, Morita E. Haemaphysalis longicornis tick bites are a possible cause of red meat allergy in Japan. Allergy. 2016;71(3):421–5. doi: 10.1111/all.12804 . 26551325 10.1111/all.12804

[11] Fischer J, Lupberger E, Hebsaker J, Blumenstock G, Aichinger E, Yazdi AS, et al Prevalence of type I sensitization to alpha-gal in forest service employees and hunters. Allergy. 2017 doi: 10.1111/all.13156 . 28273338 10.1111/all.13156

[12] Gonzalez-Quintela A, Dam Laursen AS, Vidal C, Skaaby T, Gude F, Linneberg A. IgE antibodies to alpha-gal in the general adult population: relationship with tick bites, atopy, and cat ownership. Clin Exp Allergy. 2014;44(8):1061–8. doi: 10.1111/cea.12326 . 24750173 10.1111/cea.12326

[13] Piesman J, Gern L. Lyme borreliosis in Europe and North America. Parasitology. 2004;129 Suppl:S191–220. . 15938512 10.1017/s0031182003004694

[14] Rispens T, Derksen NI, Commins SP, Platts-Mills TA, Aalberse RC. IgE production to alpha-gal is accompanied by elevated levels of specific IgG1 antibodies and low amounts of IgE to blood group B. PLoS One. 2013;8(2):e55566 doi: 10.1371/journal.pone.0055566 . 23390540 10.1371/journal.pone.0055566 PMC3563531

[15] Wilhelmsson P, Fryland L, Lindblom P, Sjowall J, Ahlm C, Berglund J, et al A prospective study on the incidence of Borrelia burgdorferi sensu lato infection after a tick bite in Sweden and on the Aland Islands, Finland (2008–2009). Ticks Tick Borne Dis. 2016;7(1):71–9. doi: 10.1016/j.ttbdis.2015.08.009 . 26341726 10.1016/j.ttbdis.2015.08.009

[16] Wilhelmsson P, Lindblom P, Fryland L, Ernerudh J, Forsberg P, Lindgren PE. Prevalence, diversity, and load of Borrelia species in ticks that have fed on humans in regions of Sweden and Aland Islands, Finland with different Lyme borreliosis incidences. PLoS One. 2013;8(11):e81433 doi: 10.1371/journal.pone.0081433 . 24278437 10.1371/journal.pone.0081433 PMC3836827

[17] Stanek G, Fingerle V, Hunfeld KP, Jaulhac B, Kaiser R, Krause A, et al Lyme borreliosis: clinical case definitions for diagnosis and management in Europe. Clin Microbiol Infect. 2011;17(1):69–79. doi: 10.1111/j.1469-0691.2010.03175.x . 20132258 10.1111/j.1469-0691.2010.03175.x

[18] Strle F, Stanek G. Clinical manifestations and diagnosis of lyme borreliosis. Curr Probl Dermatol. 2009;37:51–110. doi: 10.1159/000213070 . 19367097 10.1159/000213070

[19] Geblod.nu. Facts about blood and blood groups 2017 [cited 2017 3 April]. https://geblod.nu/fakta/?c=7.

[20] Tjernberg I, Kruger G, Eliasson I. C6 peptide ELISA test in the serodiagnosis of Lyme borreliosis in Sweden. Eur J Clin Microbiol Infect Dis. 2007;26(1):37–42. doi: 10.1007/s10096-006-0239-3 . 17180348 10.1007/s10096-006-0239-3

[21] Tjernberg I, Sillanpaa H, Seppala I, Eliasson I, Forsberg P, Lahdenne P. Antibody responses to borrelia IR(6) peptide variants and the C6 peptide in Swedish patients with erythema migrans. Int J Med Microbiol. 2009;299(6):439–46. doi: 10.1016/j.ijmm.2008.10.006 . 19138558 10.1016/j.ijmm.2008.10.006

[22] Armitage P, Berry G, JNS M. Statistical methods in medical research. 4th ed London: Blackwell; 2002.

[23] Kilpatrick AM, Dobson ADM, Levi T, Salkeld DJ, Swei A, Ginsberg HS, et al Lyme disease ecology in a changing world: consensus, uncertainty and critical gaps for improving control. Philos Trans R Soc Lond B Biol Sci. 2017;372(1722). doi: 10.1098/rstb.2016.0117 . 28438910 10.1098/rstb.2016.0117 PMC5413869

[24] Berglund J, Eitrem R, Ornstein K, Lindberg A, Ringer A, Elmrud H, et al An epidemiologic study of Lyme disease in southern Sweden. N Engl J Med. 1995;333(20):1319–27. Epub 1995/11/16. doi: 10.1056/NEJM199511163332004 . 7566023 10.1056/NEJM199511163332004

[25] Johansson M, Manfredsson L, Wistedt A, Serrander L, Tjernberg I. Significant variations in the seroprevalence of C6 ELISA antibodies in a highly endemic area for Lyme borreliosis: evaluation of age, sex and seasonal differences. APMIS. 2017 doi: 10.1111/apm.12664 . 28225145 10.1111/apm.12664

